# Blending type approximation by $GBS$ operators of bivariate tensor product of *λ*-Bernstein–Kantorovich type

**DOI:** 10.1186/s13660-018-1862-0

**Published:** 2018-10-01

**Authors:** Qing-Bo Cai, Guorong Zhou

**Affiliations:** 1 School of Mathematics and Computer Science, Quanzhou Normal University, Quanzhou, China; 2 School of Applied Mathematics, Xiamen University of Technology, Xiamen, China

**Keywords:** 41A10, 41A25, 41A36, *B*-continuous functions, *B*-differentiable functions, $GBS$ operators, Bernstein–Kantorovich operators, Mixed modulus of smoothness

## Abstract

In this paper, we introduce a family of $GBS$ operators of bivariate tensor product of *λ*-Bernstein–Kantorovich type. We estimate the rate of convergence of such operators for *B*-continuous and *B*-differentiable functions by using the mixed modulus of smoothness, establish the Voronovskaja type asymptotic formula for the bivariate *λ*-Bernstein–Kantorovich operators, as well as give some examples and their graphs to show the effect of convergence.

## Introduction

In 1912, Bernstein [1] constructed a sequence of polynomials to prove the Weierstrass approximation theorem as follows:

1 $$\begin{aligned} B_{n}(f;x)=\sum_{k=0}^{n}f \biggl( \frac{k}{n} \biggr) b_{n,k}(x), \end{aligned}$$

for any continuous function $f\in C[0,1]$, where $x\in [0,1]$, $n=1,2,\dots $, and Bernstein basis functions $b_{n,k}(x)$ are defined by

2 $$b_{n,k}(x)=\left(\begin{array}{c} n\\ k \end{array}\right)x^{k}{\left(1-x\right)}^{n-k}.$$

The polynomials in (1), called Bernstein polynomials, possess many remarkable properties.

Recently, Cai et al. [2] proposed a new type *λ*-Bernstein operators with parameter $\lambda \in [-1,1]$, they obtained some approximation properties and gave some graphs and numerical examples to show that these operators converge to continuous functions *f*. These operators, which they called *λ*-Bernstein operators, are defined as follows:

3 $$\begin{aligned} B_{n,\lambda }(f;x)=\sum_{k=0}^{n} \widetilde{b}_{n,k}(\lambda;x)f \biggl( \frac{k}{n} \biggr), \end{aligned}$$

where

4 $$\begin{aligned} \textstyle\begin{cases} \widetilde{b}_{n,0}(\lambda;x)=b_{n,0}(x)-\frac{\lambda }{n+1}b_{n+1,1}(x), \\ \widetilde{b}_{n,i}(\lambda;x)=b_{n,i}(x)+\lambda ( \frac{n-2i+1}{n ^{2}-1}b_{n+1,i}(x)-\frac{n-2i-1}{n^{2}-1}b_{n+1,i+1}(x) ), \\ \widetilde{b}_{n,n}(\lambda;x)=b_{n,n}(x)-\frac{\lambda }{n+1}b_{n+1,n}(x), \end{cases}\displaystyle \end{aligned}$$

$1\leq i\leq n-1$, ${b}_{n,k}(x)$ ($k=0,1,\dots,n$) are defined in (2) and $\lambda \in [-1,1]$.

In [3], Cai introduced the *λ*-Bernstein–Kantorovich operators as

5 $$\begin{aligned} K_{n,\lambda }(f;x)=(n+1)\sum_{k=0}^{n} \widetilde{b}_{n,k}(\lambda;x) \int_{\frac{k}{n+1}}^{\frac{k+1}{n+1}}f(t)\,dt, \end{aligned}$$

where $\widetilde{b}_{n,k}(\lambda,x)$ ($k=0,1,\dots,n$) are defined in (2) and $\lambda \in [-1,1]$. He established a global approximation theorem in terms of second order modulus of continuity, obtained a direct approximation theorem by means of the Ditzian–Totik modulus of smoothness and derived an asymptotically estimate on the rate of convergence for certain absolutely continuous functions. Very recently, Acu et al. provided a quantitative Voronovskaja type theorem, a Grüss–Voronovskaja type theorem, and also gave some numerical examples of the operators defined in (5) in [4].

As we know, the generalized Boolean sum operators (abbreviated by $GBS$ operators) were first studied by Dobrescu and Matei in [5]. The Korovkin theorem for *B*-continuous functions was established by Badea et al. in [6, 7]. In 2013, Miclăuş [8] studied the approximation by the $GBS$ operators of Bernstein–Stancu type. In 2016, Agrawal et al. [9] considered the bivariate generalization of Lupaş–Durrmeyer type operators based on Pólya distribution and studied the degree of approximation for the associated $GBS$ operators. In 2017, Bărbosu et al. [10] introduced the $GBS$ operators of Durrmeyer type based on *q*-integers, studied the uniform convergence theorem and the degree of approximation of these operators. Very recently, Kajla and Miclăuş [11] introduced the $GBS$ operators of generalized Bernstein–Durrmeyer type and estimated the degree of approximation in terms of the mixed modulus of smoothness.

Motivated by the above research, the aims of this paper are to propose the bivariate tensor product of *λ*-Bernstein–Kantorovich operators and the $GBS$ operators of bivariate tensor product of *λ*-Bernstein–Kantorovich type. We use the mixed modulus of smoothness to estimate the rate of convergence of GBS operators of bivariate tensor product of *λ*-Bernstein–Kantorovich type for *B*-continuous and *B*-differentiable functions, and establish a Voronovskaja type asymptotic formula for the bivariate *λ*-Bernstein–Kantorovich operators. In order to show the effect of convergence, we also give some examples and graphs.

This paper is mainly organized as follows: In Sect. 2, we introduce the bivariate tensor product of *λ*-Bernstein–Kantorovich operators $K_{m,n}^{\lambda_{1},\lambda_{2}}(f;x,y)$ and the $GBS$ operators $UK_{m,n}^{\lambda_{1},\lambda_{2}}(f;x,y)$. In Sect. 3, some lemmas are given to prove the main results. In Sect. 4, the rate of convergence for *B*-continuous and *B*-differentiable functions of $GBS$ operators $UK_{m,n}^{\lambda_{1},\lambda_{2}}(f;x,y)$ is proved. In Sect. 5, we investigate the Voronovskaja type asymptotic formula for bivariate operators $K_{m,n}^{\lambda_{1},\lambda_{2}}(f;x,y)$.

## Construction of operators

For $f\in C(I^{2})$, $I^{2}=[0,1]\times [0,1]$, $\lambda_{1}, \lambda _{2}\in [-1,1]$, we introduce the bivariate tensor product of *λ*-Bernstein–Kantorovich operators as

6 $$\begin{aligned}& K_{m,n}^{\lambda_{1},\lambda_{2}}(f;x,y) \\& \quad =(m+1) (n+1)\sum_{i=0}^{m}\sum _{j=0}^{n}\widetilde{b}_{m,i}( \lambda _{1};x)\widetilde{b}_{n,j}(\lambda_{2};y) \int_{\frac{i}{m+1}}^{ \frac{i+1}{m+1}} \int_{\frac{j}{n+1}}^{\frac{j+1}{n+1}}f(t,s)\,dt\,ds, \end{aligned}$$

where $\widetilde{b}_{m,i}(\lambda_{1};x)$ ($i=0,1,\dots,n$) and $\widetilde{b}_{n,j}(\lambda_{2};y)$ ($j=0,1,\dots,n$) are defined in (4), $\lambda_{1}, \lambda_{2}\in [-1,1]$. Obviously, when $\lambda_{1}=\lambda_{2}=0$, $B_{m,n}^{0,0}(f;x,y)$ reduce to the bivariate tensor product of classical Bernstein–Kantorovich operators.

The $GBS$ operators of the bivariate tensor product of *λ*-Bernstein–Kantorovich type are defined as

7 $$\begin{aligned}& UK_{m,n}^{\lambda_{1},\lambda_{2}} \bigl( f(t,s);x,y \bigr) \\& \quad =K_{m,n}^{\lambda_{1},\lambda_{2}} \bigl( f(x,s)+f(t,y)-f(t,s);x,y \bigr) \\& \quad =(m+1) (n+1)\sum_{i=0}^{m}\sum _{j=0}^{n}\widetilde{b}_{m,i}( \lambda _{1};x)\widetilde{b}_{n,j}(\lambda_{2};y) \int_{\frac{i}{m+1}}^{ \frac{i+1}{m+1}} \int_{\frac{j}{n+1}}^{\frac{j+1}{n+1}}\bigl[ f(x,s)+f(t,y) \\& \qquad{}-f(t,s)\bigr]\,ds\,dt, \end{aligned}$$

for $f\in C_{b}(I^{2})$. Obviously, the operators $UK_{m,n}^{\lambda _{1},\lambda_{2}}(f;x,y)$ are positive linear operators.

## Auxiliary results

In order to obtain the main results, we need the following lemmas.

### Lemma 3.1

([4])

*For* *λ*-*Bernstein–Kantorovich operators* $K_{n,\lambda }(f;x)$ *and* $n>1$, *we have the following equalities*:

$$\begin{aligned}& K_{n,\lambda }(1;x) =1; \\& K_{n,\lambda }(t;x) =x+\frac{1-2x}{2(n+1)}+\frac{1-2x+x^{n+1}-(1-x)^{n+1}}{n ^{2}-1}\lambda; \\& K_{n,\lambda }\bigl(t^{2};x\bigr) =x^{2}- \frac{9nx^{2}-6nx+3x^{2}-1}{3(n+1)^{2}}+\frac{2(-2x^{2}n+x^{n+1}n+xn+x ^{n+1}-x)\lambda }{(n-1)(n+1)^{2}}; \\& K_{n,\lambda }\bigl(t^{3};x\bigr) =x^{3}- \frac{24n^{2}x^{3}-18n^{2}x^{2}+4nx ^{3}+18nx^{2}+4x^{3}-14nx-1}{4(n+1)^{3}} \\& \hphantom{K_{n,\lambda }(t^{3};x) =}{}+\frac{\lambda }{2(n+1)^{3}(n-1)}\bigl[ -12n^{2}x^{3}+6n^{2}x^{2}+12x ^{3}n+6x^{n+1}n^{2}-30x^{2}n \\& \hphantom{K_{n,\lambda }(t^{3};x) =}{} +12x^{n+1}n+6xn+7x^{n+1}-(1-x)^{n+1}-8x+1 \bigr] ; \\& K_{n,\lambda }\bigl(t^{4};x\bigr) =\frac{1}{5(n+1)^{4}} \bigl( 5n^{5}x^{4}-30n ^{3}x^{4}+40n^{3}x^{3}+55n^{2}x^{4}-120n^{2}x^{3}-30nx^{4} \\& \hphantom{K_{n,\lambda }(t^{4};x)=}{} +75n^{2}x^{2}+80nx^{3}-75nx^{2}+30nx+1 \bigr) +\frac{2\lambda }{(n-1)(n+1)^{4}}\bigl( -4n^{3}x^{4} \\& \hphantom{K_{n,\lambda }(t^{4};x)=}{} +2n^{3}x^{3}+12n^{2}x^{4}-24n^{2}x^{3} -8x^{4}n+2x^{n+1}n^{3}+6n ^{2}x^{2}+22x^{3}n \\& \hphantom{K_{n,\lambda }(t^{4};x)=}{} +6x^{n+1}n^{2}-24x^{2}n+3xn+3x^{n+1}-3x \bigr). \end{aligned}$$

### Lemma 3.2

([4])

*For* *λ*-*Bernstein–Kantorovich operators* $K_{n,\lambda }(f;x)$ *and* $n>1$, *we have*

$$\begin{aligned}& K_{n,\lambda }(t-x;x) =\frac{1-2x}{2(n+1)}+\frac{1-2x+x^{n+1}-(1-x)^{n+1}}{n ^{2}-1}\lambda; \\& K_{n,\lambda } \bigl( (t-x)^{2};x \bigr) =\frac{x(1-x)}{n+1}+ \frac{1-6x+6x ^{2}}{3(n+1)^{2}}+\frac{2\lambda [ x^{n+1}(1-x)+x(1-x)^{n+1} ] }{n ^{2}-1} \\& \hphantom{K_{n,\lambda } ( (t-x)^{2};x )=}{}-\frac{4x(1-x)\lambda }{(n+1)^{2}(n-1)}. \end{aligned}$$

### Lemma 3.3

(See [4, Lemma 2.4])

*We have*

$$\begin{aligned}& \lim_{n\rightarrow \infty }nK_{n,\lambda }(t-x;x)=\frac{1}{2}-x; \\& \lim_{n\rightarrow \infty }nK_{n,\lambda } \bigl( (t-x)^{2};x \bigr) =x(1-x),\quad x\in (0,1), \\& \lim_{n\rightarrow \infty }n^{2}K_{n,\lambda } \bigl( (t-x)^{4};x \bigr) =O(1),\quad x\in (0,1). \end{aligned}$$

### Lemma 3.4

*For the bivariate tensor product of* *λ*-*Bernstein–Kantorovich operators* $K_{m,n}^{\lambda_{1},\lambda_{2}}(f;x,y)$, *we have the following inequalities*:

$$\begin{aligned}& K_{m,n}^{\lambda_{1},\lambda_{2}} \bigl( (t-x)^{2};x,y \bigr) \leq \frac{2}{m+1}; \\& K_{m,n}^{\lambda_{1},\lambda_{2}} \bigl( (s-y)^{2};x,y \bigr) \leq \frac{2}{n+1}; \\& K_{m,n}^{\lambda_{1},\lambda_{2}} \bigl( (t-x)^{2}(s-y)^{2};x,y \bigr) \leq \frac{4}{(m+1)(n+1)}; \\& K_{m,n}^{\lambda_{1},\lambda_{2}} \bigl( (t-x)^{4}(s-y)^{2};x,y \bigr) \leq \frac{C}{(m+1)^{2}(n+1)}; \\& K_{m,n}^{\lambda_{1},\lambda_{2}} \bigl( (t-x)^{2}(s-y)^{4};x,y \bigr) \leq \frac{C}{(m+1)(n+1)^{2}}, \end{aligned}$$

*where* *C* *is a positive constant*.

## Rate of convergence

We first introduce the definitions of *B*-continuity and *B*-differentiability, details can be found in [12] and [13]. Let *X* and *Y* be compact real intervals. A function *f*: $X\times Y\rightarrow \mathbb{R}$ is called a *B*-continuous function at $(x_{0}, y_{0})\in X\times Y$ if

$$\begin{aligned} \lim_{(x,y)\rightarrow (x_{0},y_{0})}\triangle f\bigl((x,y),(x_{0},y_{0}) \bigr)=0, \end{aligned}$$

where $\triangle f((x,y),(x_{0},y_{0}))=f(x,y)-f(x_{0},y)-f(x,y_{0})+f(x _{0},y_{0})$ denotes the mixed difference of *f*. A function $f: X\times Y\rightarrow \mathbb{R}$ is a *B*-differentiable function at $(x_{0},y_{0})\in X\times Y$ if the following limit exists and is finite:

$$\begin{aligned} \lim_{(x,y)\rightarrow (x_{0},y_{0})}\frac{\triangle f((x,y),(x_{0},y _{0}))}{(x-x_{0})(y-y_{0})}. \end{aligned}$$

The limit is named the *B*-differential of *f* at the point $(x_{0},y_{0})$ and denoted by $D_{B}f(x_{0},y_{0})$.

The function $f: X\times Y\rightarrow \mathbb{R}$ is *B*-bounded on $X\times Y$ if there exists a $k>0$ such that $|\triangle f((x,y),(t,s))| \leq K$ for any $(x,y), (t,s)\in X\times Y$.

Let $B(X\times Y)$, $C(X\times Y)$ denote the spaces of all bounded functions and of all continuous functions on $X\times Y$ endowed with the sup-norm $\|\cdot \|_{\infty }$, respectively. We also define the following function sets:

$$ B_{b}(X\times Y)=\{f: X\times Y\rightarrow \mathbb{R}|f \text{ is } B \text{-bounded on } X \times Y\} $$

with the norm $\|f\|_{B}=\sup_{(x,y),(t,s)\in X\times Y}|\triangle f((x,y),(t,s))|$,

$$ C_{b}(X\times Y)=\{f: X\times Y\rightarrow \mathbb{R}|f \text{ is } B \text{-continuous on } X\times Y\}, $$

and $D_{b}(X\times Y)=\{f: X\times Y\rightarrow \mathbb{R}|f \text{ is } B\text{-differentiable on } X\times Y\}$. It is known that $C(X\times Y)\subset C_{b}(X\times Y)$.

Let $f\in B_{b}(X\times Y)$. Then the mixed modulus of smoothness $\omega_{\mathrm{mixed}}(f;\cdot,\cdot)$ is defined by

$$\begin{aligned} \omega_{\mathrm{mixed}}(f;\delta_{1},\delta_{2})=\sup \bigl\{ \bigl\vert \triangle f\bigl((x,y),(t,s)\bigr) \bigr\vert : \vert x-t \vert \leq \delta_{1}, \vert y-s \vert \leq \delta_{2} \bigr\} , \end{aligned}$$

for any $\delta_{1},\delta_{2}\geq 0$.

Let $L: C_{b}(X\times Y)\rightarrow B(X\times Y)$ be a linear positive operator. The operator $UL: C_{b}(X\times Y)\rightarrow B(X\times Y)$ defined for any function $f\in C_{b}(X\times Y)$ and any $(x,y)\in X \times Y$ by $UL ( f(t,s);x,y ) =L ( f(t,y)+f(x,s)-f(t,s);x,y ) $ is called the $GBS$ operator associated to the operator *L*.

In the sequel, we will consider functions $e_{ij}: X\times Y\rightarrow \mathbb{R}$, $e_{ij}(x,y)=x^{i}y^{j}$ for any $(x,y)\in X\times Y$, and $i,j\in \mathbb{N}$. In order to estimate the rate of convergence of $UK_{m,n}^{\lambda_{1},\lambda_{2}}(f;x,y)$, we need the following two theorems.

### Theorem 4.1

([7])

*Let* $L: C_{b}(X\times Y)\rightarrow B(X\times Y)$ *be a linear positive operator and* $UL: C_{b}(X\times Y)\rightarrow B(X\times Y)$ *the associated GBS operator*. *Then for any* $f\in C_{b}(X\times Y)$, *any* $(x,y)\in (X\times Y)$ *and* $\delta_{1},\delta_{2}>0$, *we have*

$$\begin{aligned}& \bigl\vert UL \bigl( f(t,s);x,y \bigr) -f(x,y) \bigr\vert \\& \quad \leq \bigl\vert f(x,y) \bigr\vert \bigl\vert 1-L ( e_{00};x,y ) \bigr\vert + \bigl[ L ( e_{00};x,y ) + \delta_{1}^{-1}\sqrt{L \bigl( (t-x)^{2};x,y \bigr) } \\& \qquad {} +\delta_{2}^{-1}\sqrt{L \bigl( (s-y)^{2};x,y \bigr) }+\delta _{1}^{-1}\delta_{2}^{-1} \sqrt{L \bigl( (t-x)^{2}(s-y)^{2};x,y \bigr) }\bigr] \omega_{\mathrm{mixed}}(f;\delta_{1},\delta_{2}). \end{aligned}$$

### Theorem 4.2

([14])

*Let* $L: C_{b}(X\times Y)\rightarrow B(X\times Y)$ *be a linear positive operator and* $UL: C_{b}(X\times Y)\rightarrow B(X\times Y)$ *the associated GBS operator*. *Then for any* $f\in D_{b}(X\times Y)$ *with* $D_{B}f\in B(X\times Y)$, *any* $(x,y)\in (X\times Y)$ *and* $\delta_{1}, \delta_{2}>0$, *we have*

$$\begin{aligned}& \bigl\vert UL \bigl( f(t,s);x,y \bigr) -f(x,y) \bigr\vert \\& \quad \leq \bigl\vert f(x,y) \bigr\vert \bigl\vert 1-L ( e_{00};x,y ) \bigr\vert +3 \Vert D_{B}f \Vert _{ \infty }\sqrt{L \bigl( (t-x)^{2}(s-y)^{2};x,y \bigr) } \\& \qquad {}+ \bigl[ \sqrt{L \bigl( (t-x)^{2}(s-y)^{2};x,y \bigr) }+\delta_{1}^{-1}\sqrt{L \bigl( (t-x)^{4}(s-y)^{2};x,y \bigr) } \\& \qquad {}+\delta_{2}^{-1}\sqrt{L \bigl( (t-x)^{2}(s-y)^{4};x,y \bigr) }+ \delta_{1}^{-1} \delta_{2}^{-1}L \bigl( (t-x)^{2}(s-y)^{2};x,y \bigr) \bigr] \\& \qquad {}\times \omega_{\mathrm{mixed}} ( D_{B}f;\delta_{1}, \delta_{2} ). \end{aligned}$$

First, we will use *B*-continuous functions to estimate the rate of convergence of $UK_{m,n}^{\lambda_{1},\lambda_{2}}(f;x,y)$ to $f\in C_{b}(I^{2})$ by using the mixed modulus of smoothness. We have

### Theorem 4.3

*For* $f\in C_{b}(I^{2})$, $(x,y)\in I^{2}$ *and* $m,n>1$, *we have the following inequality*:

8 $$\begin{aligned} \bigl\vert UK_{m,n}^{\lambda_{1},\lambda_{2}}(f;x,y)-f(x,y) \bigr\vert \leq ( 3+2\sqrt{2} ) \omega_{\mathrm{mixed}} \biggl( f; \frac{1}{\sqrt{m+1}}, \frac{1}{\sqrt{n+1}} \biggr). \end{aligned}$$

### Proof

Applying Theorem 4.1 and using Lemma 3.4, we get

$$\begin{aligned}& \bigl\vert UK_{m,n}^{\lambda_{1},\lambda_{2}}(f;x,y)-f(x,y) \bigr\vert \\& \quad \leq \biggl[ 1+\frac{1}{\delta_{1}}\sqrt{\frac{2}{m+1}}+ \frac{1}{ \delta_{2}}\sqrt{\frac{2}{n+1}}+\frac{2}{\delta_{1}\delta_{2} \sqrt{(m+1)(n+1)}} \biggr] \omega_{\mathrm{mixed}}(f;\delta_{1},\delta_{2}). \end{aligned}$$

Therefore, (8) can be obtained from the above inequality by choosing $\delta_{1}=\frac{1}{\sqrt{m+1}}$ and $\delta_{2}=\frac{1}{ \sqrt{n+1}}$. □

Next, we will give the rate of convergence to the *B*-differentiable functions for $UK_{m,n}^{\lambda_{1},\lambda_{2}}(f;x,y)$.

### Theorem 4.4

*Let* $f\in D_{b}(I^{2})$, $D_{B}f\in B(I^{2})$, $(x,y)\in I^{2}$ *and* $m,n>1$, *we have the following inequality*:

9 $$\begin{aligned}& \bigl\vert UK_{m,n}^{\lambda_{1},\lambda_{2}}(f;x,y)-f(x,y) \bigr\vert \\& \quad \leq \frac{M}{\sqrt{(m+1)(n+1)}} \biggl[ \Vert D_{B}f \Vert _{\infty }+ \omega_{\mathrm{mixed}} \biggl( D_{B}f; \frac{1}{\sqrt{m+1}},\frac{1}{ \sqrt{n+1}} \biggr) \biggr] , \end{aligned}$$

*where* *C* *and* *M* *are positive constants*.

### Proof

Using Theorem 4.2 and Lemma 3.4, we have

$$\begin{aligned}& \bigl\vert UK_{m,n}^{\lambda_{1},\lambda_{2}}(f;x,y)-f(x,y) \bigr\vert \\& \quad \leq \frac{6 \Vert D_{B}f \Vert _{\infty }}{\sqrt{(m+1)(n+1)}}+ \biggl[ \frac{2}{ \sqrt{(m+1)(n+1)}}+\frac{1}{\delta_{1}(m+1)} \sqrt{\frac{C}{n+1}} \\& \qquad {} +\frac{1}{\delta_{2}(n+1)}\sqrt{\frac{C}{m+1}}+\frac{4}{ \delta_{1}\delta_{2}(m+1)(n+1)} \biggr] \omega_{\mathrm{mixed}} ( D_{B}f; \delta_{1}, \delta_{2} ). \end{aligned}$$

Hence, taking $\delta_{1}=\frac{1}{\sqrt{m+1}}$, $\delta_{2}=\frac{1}{ \sqrt{n+1}}$ and using the above inequality, we get the desired result (9). □

### Example 4.5

**Figure 1:**
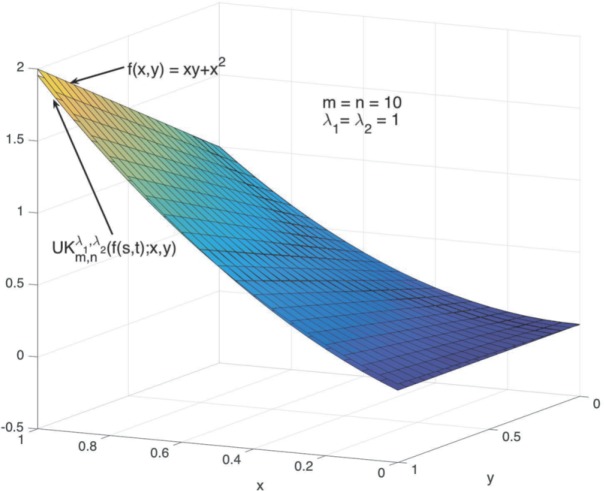
Graphs of $UK_{10,10}^{1,1}(f(s,t);x,y)$ and $f(x,y)$

**Figure 2:**
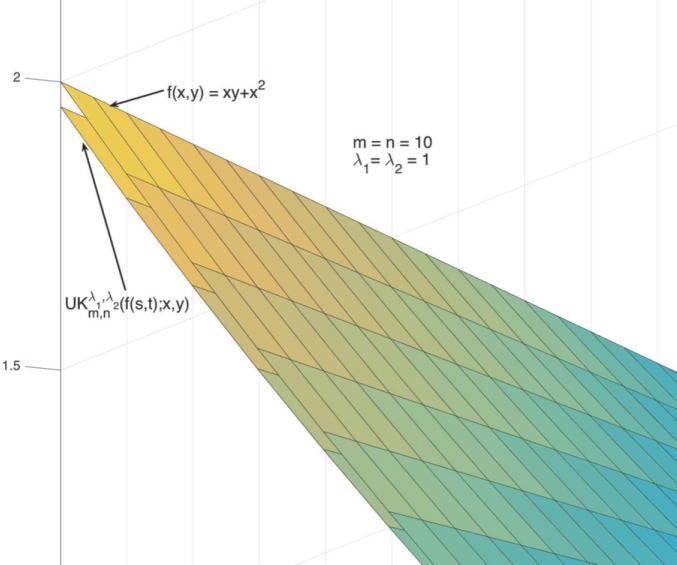
Partially enlarged graphs of $UK_{10,10}^{1,1}(f(s,t);x,y)$ and $f(x,y)$

Let $f(x,y) = xy+x^{2}$, $x,y\in [0,1]$, the graphs of $f(x,y)$ and $UK_{10,10}^{1,1}(f(s,t);x,y)$ are shown in Fig. 1. Figure 2 shows the partially enlarged graphs of $f(x,y)$ and $UK_{10,10}^{1,1}(f(s,t);x,y)$.

## Voronovskaja type asymptotic formulas for $K_{m,n}^{\lambda _{1},\lambda _{2}}(f;x,y)$

In this section, we will give a Voronovskaja type asymptotic formula for $K_{m,n}^{\lambda_{1},\lambda_{2}}(f;x,y)$.

### Theorem 5.1

*Consider an* $f\in C(I^{2})$. *Then for any* $x,y\in (0,1)$ *and* $\lambda_{1},\lambda_{2}\in [-1,1]$, *we have*

$$\begin{aligned}& \lim_{n\rightarrow \infty }n \bigl[ K_{n,n}^{\lambda_{1},\lambda_{2}}(f;x,y)-f(x,y) \bigr] \\& \quad =\frac{f^{\prime}_{x}(x,y)}{2}(1-2x)+\frac{f^{\prime}_{y}(x,y)}{2}(1-2y)+ \frac{1}{2} \bigl[ f^{\prime\prime }_{x^{2}}(x,y)x(1-x)+f^{\prime\prime }_{y^{2}}(x,y)y(1-y) \bigr] . \end{aligned}$$

### Proof

For $(x,y), (t,s)\in I^{2}$, by Taylor’s expansion, we have

10 $$\begin{aligned}& f(t,s) \\& \quad =f(x,y)+f^{\prime}_{x}(x,y) (t-x)+f^{\prime}_{y}(x,y) (s-y)+\frac{1}{2} \bigl[ f ^{\prime\prime }_{x^{2}}(x,y) (t-x)^{2}+2f^{\prime\prime }_{xy}(x,y) \\& \qquad {}\times (t-x) (s-y)+f^{\prime\prime }_{y^{2}}(x,y) (s-y)^{2} \bigr] +\rho (t,s;x,y) \sqrt{(t-x)^{4}+(s-y)^{4}}, \end{aligned}$$

where $\rho (t,s;x,y)\in C(I^{2})$ and $\lim_{(t,s)\rightarrow (x,y)} \rho (t,s;x,y)=0$.

Applying $K_{n,n}^{\lambda_{1},\lambda_{2}}(f;x)$ to (10), we obtain

$$\begin{aligned} K_{n,n}^{\lambda_{1},\lambda_{2}}(f;x,y) =&f(x,y)+f^{\prime}_{x}(x,y)K _{n,\lambda_{1}}(t-x;x)+f^{\prime}_{y}(x,y)K_{n,\lambda_{2}}(s-y;y) \\ &{}+\frac{1}{2} \bigl[ f^{\prime\prime }_{x^{2}}(x,y)K_{n,\lambda_{1}} \bigl( (t-x)^{2};x \bigr) +f ^{\prime\prime }_{y^{2}}(x,y)K_{n,\lambda_{2}} \bigl( (s-y)^{2};y \bigr) \\ &{} +2f^{\prime\prime }_{xy}(x,y)K_{n,n}^{\lambda_{1},\lambda_{2}} \bigl( (t-x) (s-y);x,y \bigr) \bigr] \\ &{}+K_{n,n}^{\lambda_{1},\lambda_{2}} \bigl( \rho (t,s;x,y) \sqrt{(t-x)^{4}+(s-y)^{4}};x,y \bigr). \end{aligned}$$

Taking the limit on both sides of the above equality, we have

11 $$\begin{aligned}& \lim_{n\rightarrow \infty }n \bigl[ K_{n,n}^{\lambda_{1},\lambda_{2}}(f;x,y)-f(x,y) \bigr] \\& \quad =f^{\prime}_{x}(x,y)\lim_{n\rightarrow \infty }nK_{n,\lambda_{1}}(t-x;x)+f ^{\prime}_{y}(x,y)\lim_{n\rightarrow \infty }nK_{n,\lambda_{2}}(s-y;y) \\& \qquad {}+\frac{1}{2} \Bigl[ f^{\prime\prime }_{x^{2}}(x,y)\lim _{n\rightarrow \infty }nK _{n,\lambda_{1}} \bigl( (t-x)^{2};x \bigr) \\& \qquad {}+f^{\prime\prime }_{y^{2}}(x,y)\lim_{n\rightarrow \infty }nK_{n,\lambda_{2}} \bigl( (s-y)^{2};y \bigr) \\& \qquad {}+ 2f^{\prime\prime }_{xy}(x,y)\lim_{n\rightarrow \infty }nK_{n,n}^{\lambda _{1},\lambda_{2}} \bigl( (t-x) (s-y);x,y \bigr) \Bigr] \\& \qquad {}+\lim_{n\rightarrow \infty }nK_{n,n}^{\lambda_{1},\lambda_{2}} \bigl( \rho (t,s;x,y) \sqrt{(t-x)^{4}+(s-y)^{4}};x,y \bigr). \end{aligned}$$

Using Lemma 3.2, we have

12 $$\begin{aligned}& \lim_{n\rightarrow \infty }nK_{n,n}^{\lambda_{1},\lambda_{2}} \bigl( (t-x) (s-y);x,y \bigr) =\lim_{n\rightarrow \infty }n \bigl[ K_{n,\lambda_{1}}(t-x;x)K_{n, \lambda_{2}}(s-y;y) \bigr] =0. \end{aligned}$$

By Cauchy–Schwarz inequality, we have

$$\begin{aligned}& nK_{n,n}^{\lambda_{1},\lambda_{2}} \bigl( \rho (t,s;x,y) \sqrt{(t-x)^{4}+(s-y)^{4}};x,y \bigr) \\& \quad \leq \sqrt{K_{n,n}^{\lambda_{1},\lambda_{2}} \bigl( \rho^{2}(t,s;x,y);x,y \bigr) }\sqrt{n ^{2}K_{n,n}^{\lambda_{1},\lambda_{2}} \bigl( (t-x)^{4}+(s-y)^{4};x,y \bigr) } \\& \quad \leq \sqrt{K_{n,n}^{\lambda_{1},\lambda_{2}} \bigl( \rho^{2}(t,s;x,y);x,y \bigr) } \\& \qquad {}\times \sqrt{n^{2}K_{n,\lambda_{1}} \bigl( (t-x)^{4};x \bigr) +n^{2}K _{n,\lambda_{2}} \bigl( (s-y)^{4};y \bigr) }. \end{aligned}$$

Since $\lim_{(t,s)\rightarrow (x,y)}\rho (t,s;x,y)=0$, using Lemma 3.3, we obtain

13 $$\begin{aligned} \lim_{n\rightarrow \infty }nK_{n,n}^{\lambda_{1},\lambda_{2}} \bigl( \rho (t,s;x,y) \sqrt{(t-x)^{4}+(s-y)^{4}};x,y \bigr) =0. \end{aligned}$$

Therefore, by (11), (12), (13) and Lemma 3.3, we have

$$\begin{aligned}& \lim_{n\rightarrow \infty }n \bigl[ K_{n,n}^{\lambda_{1},\lambda_{2}}(f;x,y)-f(x,y) \bigr] \\& \quad =\frac{f^{\prime}_{x}(x,y)}{2}(1-2x)+\frac{f^{\prime}_{y}(x,y)}{2}(1-2y)+ \frac{1}{2} \bigl[ f^{\prime\prime }_{x^{2}}(x,y)x(1-x)+f^{\prime\prime }_{y^{2}}(x,y)y(1-y) \bigr] . \end{aligned}$$

Thus we have obtained the desired result. □

### Example 5.2

**Figure 3:**
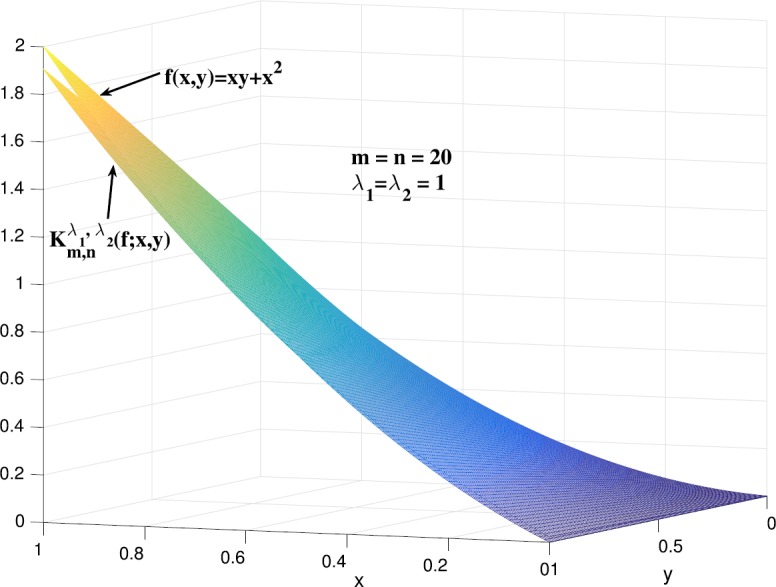
Graphs of $K_{20,20}^{1,1}(f;x,y)$ and $f(x,y)$

**Figure 4:**
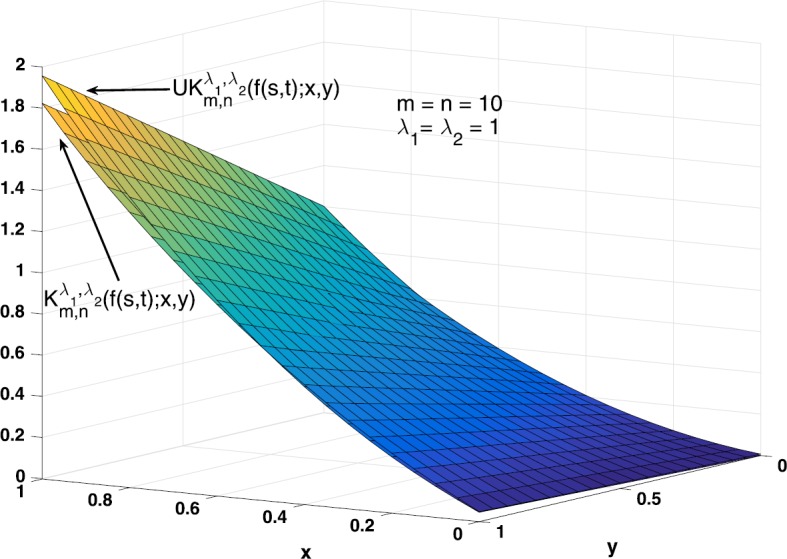
Graphs of $K_{10,10}^{1,1}(f;x,y)$ and $UK_{10,10}^{1,1}(f(s,t);x,y)$

Consider the function $f(x,y) = xy+x^{2}$, $x,y\in [0,1]$. The graphs of $f(x,y)$ and $K_{20,20}^{1,1}(f;x,y)$ are shown in Fig. 3. We also give the graphs of $K_{10,10}^{1,1}(f;x,y)$ and $UK_{10,10}^{1,1}(f(s,t);x,y)$ in Fig. 4 to compare the bivariate *λ*-Bernstein–Kantorovich operators with GBS operators.

## Conclusion

In this paper, we deduce the rate of convergence of GBS operators of bivariate tensor product of *λ*-Bernstein–Kantorovich type for *B*-continuous and *B*-differentiable functions by using the mixed modulus of smoothness, as well as obtain the Voronovskaja type asymptotic formula for bivariate *λ*-Bernstein–Kantorovich operators.
